# Influence of the Curve of Spee on Tooth Displacement Patterns: A Finite Element Analysis at Varying Implant Heights

**DOI:** 10.7759/cureus.54283

**Published:** 2024-02-16

**Authors:** Pankaj K Pareek, Ashish Kumar, Bhanwar S Bhariya, Renuka Bamal, Lucky Yadav, Akanksha Jaswal

**Affiliations:** 1 Department of Orthodontics, Rajasthan Dental College and Hospital, Jaipur, IND; 2 Orthodontist, Balaji Align and Smile Care, Jaipur, IND; 3 Department of Orthodontics, NIMS Dental College and Hospital, Jaipur, IND; 4 Department of Dental and Oral Surgery, Lady Hardinge Medical College, Delhi, IND; 5 Orthodontist, My Smile Dental Clinic, Jaipur, IND

**Keywords:** orthodontic anchorage, tooth displacement, finite element method (fem), mini-screws, curve of spee (cos)

## Abstract

Background

Monocortical mini-screw-type temporary anchorage devices (TADs), or mini-screws, have significantly impacted orthodontic treatment strategies, especially in severe crowding and protrusion cases. These devices offer flexibility in placement sites, but the chosen location can considerably influence tooth displacement patterns. Key factors include the 'line of force' and the biomechanical properties of orthodontic tools. By analyzing tension distribution and three-dimensional displacements, the finite element method (FEM) provides a thorough means to comprehend these patterns. The Curve of Spee (COS) is a crucial factor potentially affecting displacement.

Objective

This study aimed to leverage finite element analysis (FEA) to understand the impact of varying mini-implant heights (10 mm, 13 mm, and 16 mm) on the displacements of different tooth types under a consistent force of 150 gm and compare these displacements both in the presence and absence of the COS.

Materials and methods

A CAD model of the jaw and teeth was developed using CT scan data and a Rexcan III 3D White Light Scanner. This model was meshed in Altair HyperMesh using tetrahedral elements, resulting in a Finite Element Model. The model incorporated various components, including teeth, the periodontal ligament (PDL), alveolar bone, brackets, a titanium mini-screw, and an archwire measuring 0.019 x 0.025 inches. Unique material properties were assigned to the PDL, and the assembly accurately replicated the clinical alignment of the archwire and brackets. Subsequently, stress and strain analyses were conducted on the model using the FEM.

Results

The displacement patterns of various teeth at implant heights of 10 mm, 13 mm, and 16 mm under a 150-gm force were analyzed in relation to the COS. Notably, for the central incisor, the COS significantly affected displacements in the Y and Z directions. Similarly, the Lateral Incisor and Canine exhibited marked changes in the Z direction with the presence of the COS. The Second Premolar's apex displacement showed significant variation due to the COS, while the First Molar displayed notable changes in the X direction. Generally, the presence of the COS either maintained or slightly increased Z-directional displacements across teeth, particularly at the apices.

Conclusion

The presence of COS significantly influences tooth displacement patterns when using mini-screws at different implant heights. Central incisors, lateral incisors, and canines are particularly sensitive to changes in the Z direction with the COS. The biomechanical analysis emphasizes the importance of considering COS in treatment planning for optimal results with mini-implants in orthodontics.

## Introduction

Temporary anchorage devices (TADs) of the monocortical mini-screw type, commonly known as mini-screws, have gained popularity in orthodontics for securing anchorage, especially in cases of extensive crowding and protrusion that require premolar extraction [1]. Patients with vertical aesthetic issues, such as a deep bite or a gummy smile, often need anterior retraction through specific techniques. Mini-screws offer various insertion options, including the interradicular location involving the second premolar and the first molar for anterior segment retraction following premolar extraction and the subapical area for incisor intrusion [2,3].

The displacement pattern of molars can be significantly influenced by force vectors, making the placement location crucial. Establishing a connection between the mini-screw head and attachment hooks creates a 'line of force,' facilitating anterior tooth advancement by channeling the force through the center of resistance of the teeth. This center is modified by the placement of the mini-implants along the overall length of the anterior retraction (ARH) [4,5]. In the field of orthodontics, a fundamental understanding of biomechanics is essential, and biomechanical evaluation of orthodontic instruments must precede any treatments. The sophisticated numerical approach known as the finite element method (FEM) is capable of performing stress distribution analysis as well as three-dimensional (3D) displacement analysis on complex structures [6,7]. In FEM, the object is simulated as a mesh of elements connected at nodes with specified material properties. This mathematical model provides information regarding the object's stresses, strains, and displacements [7,8].

The FEM was employed due to its flexibility in applying different force systems at various locations and angles, allowing for the analysis of how these forces were distributed along the wire and its supporting components. In the current study, FEA was utilized to examine how several factors influenced the development of an orthodontic mini-implant-anchored retraction system for mass patient treatment. Using a constant force of 150 gm and three distinct implant heights (10 mm, 13 mm, and 16 mm), this study compared the X, Y, and Z axis displacements of six different tooth types (central incisor, lateral incisor, canine, second premolar, first molar, and second molar) with and without the presence of the Curve of Spee. The primary objective of this research is to illuminate the cranio-occlusal system's influence on the patterns of tooth displacement and to better understand the potential implications of these effects on the position and reliability of dental implants.

## Materials and methods

Finite element method (FEM)

This approach was employed to investigate the effects of strain and stress on living structures. This process involves constructing a network of discrete components that effectively captures the geometric characteristics of the object under consideration. The points where the mesh intersects are commonly referred to as nodes. These nodes are interconnected with the objective of establishing a cohesive system of constituent parts. The determination of the distortion experienced by individual components of the mesh under the influence of an applied force can be achieved by acquiring knowledge of the mechanical characteristics of the item, including the modulus of elasticity and Poisson's ratio. This approach allows for a detailed analysis of how strain and stress are distributed throughout the structure.

Generation of CAD model

**Figure 1:**
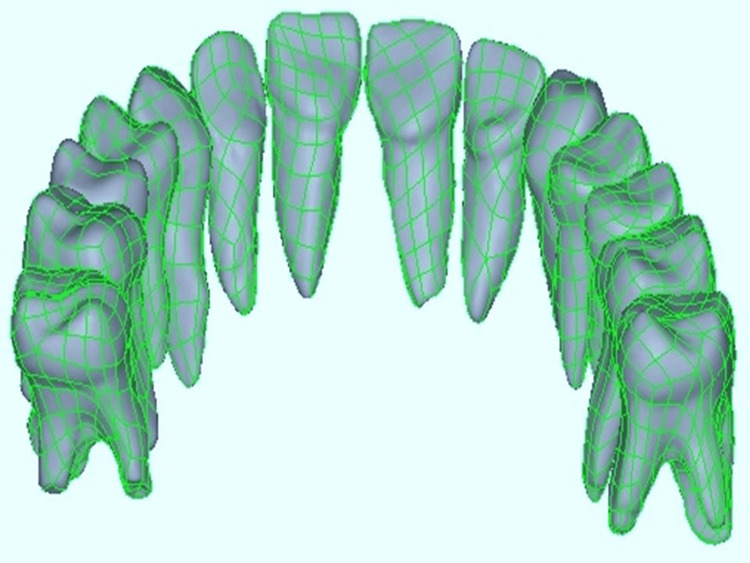
Generation of CAD Model Authors own work.

The mandible bone and dentition were modeled using Computer-Aided Design (CAD). Acquiring a CT scan of a typical human jaw, complete with the alveolar bone and teeth, was necessary for the process. CBCT.STL files were exported from the scanning center and incorporated into Altair HyperMesh to generate a virtual 3D CAD model. Using a Rexcan III 3D White Light Scanner (SOLUTIONIX), the physical typodontic model was 3D scanned. Utilizing Geomagic Modelling Software (3D Systems, North Carolina), the scanned data was employed to construct the geometric model of the tooth with bonded retainers. The crown length was determined through scanning, while the origins of the molars were fabricated using dimensions from Wheeler's textbook (Figure 1).

Meshing

**Figure 2:**
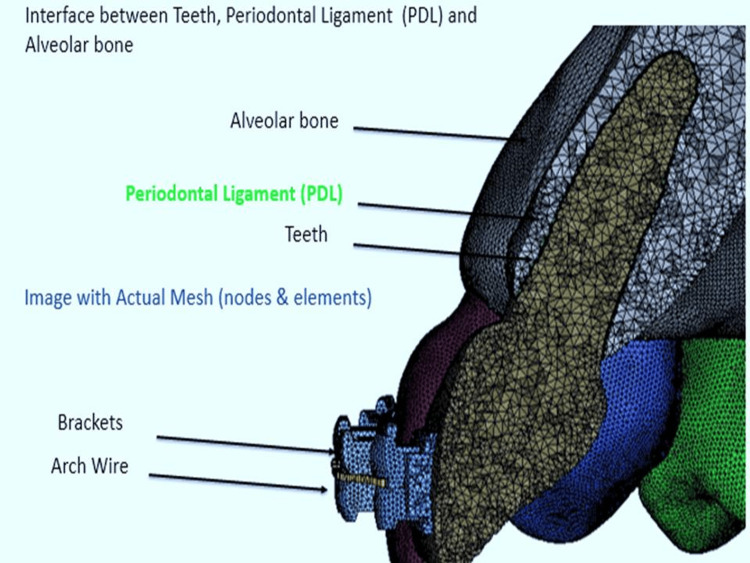
Meshing details, an interface between teeth, periodontal ligament (PDL), and alveolar bone Author's own work.

Through a procedure called 'meshing,' the CAD model was transformed into a 'Finite Element Model.' To convert the CAD model into a FE model, tetrahedral elements were added. The finite element mesh for the mandible, dentition, periodontal ligament (PDL), screw, and archwire was generated using both Hexahedral and Tetrahedral elements. The wire cross-section was created using a square measuring 0.019 x 0.025 inches (Figure 2).

Finite element model assembly

The finite element model comprised teeth, PDL, cortical and alveolar bone, brackets, titanium micro-screws, and archwire. The arch wires and brackets were positioned in accordance with the doctor's prescribed jaw alignment. The brackets were affixed to the surfaces of the teeth, following established clinical protocols. The PDL, a thin tissue layer that connects teeth to the surrounding bone, was represented in the model using distinct material characteristics.

Boundary conditions and analysis

The final model ensured the proper alignment of the brackets as per the prescription. Subsequently, the model underwent boundary conditions and was solved using FEM for stress and strain analysis.

## Results

Table 1 presents the displacements of different types of teeth in the X, Y, and Z directions, considering a 10 mm implant height under the influence of a 150-gm force. The analysis of these displacements is conducted in both the presence and absence of the Curve of Spee (COS). The most noticeable modifications caused by the COS for the Central Incisor are those occurring in the Y and Z directions. The crown demonstrates increased displacement in the Y direction, particularly when accounting for the curvature. The Lateral Incisor follows a similar pattern, except that its apex undergoes a more significant change in the Z direction as the curve progresses.

**Table 1:** Displacement in X,Y,Z direction at 10 millimetre (mm) implant height and 150 gm force

Type of Teeth	Without Curve of Spee	With Curve of Spee
Central Incisor		
Crown (X,Y,Z)	0.000000, 0.000140, -0.000026	0.000000, 0.000120, 0.000035
Apex (X,Y,Z)	0.000000, 0.000014, 0.000045	0.000000, 0.000039, 0.000078
Lateral Incisor		
Crown (X,Y,Z)	0.000001, 0.000130, 0.000026	-0.000001, 0.000120, 0.000038
Apex (X,Y,Z)	0.000008, 0.000009, 0.000039	0.000016, 0.000032, 0.000075
Canine		
Crown (X,Y,Z)	0.000004, 0.000140, 0.000025	0.000000, 0.000130, 0.000041
Apex (X,Y,Z)	-0.000004, 0.000001, 0.000030	0.000006, 0.000028, 0.000071
Second Premolar		
Crown (X,Y,Z)	0.000007, 0.000120, 0.000015	0.000001, 0.000110, 0.000054
Apex (X,Y,Z)	-0.000007,- 0.000008, 0.000009	0.000006, 0.000010, 0.000068
First Molar		
Crown (X,Y,Z)	0.000001, 0.0000130, 0.000015	-0.000003, 0.000110, 0.000053
Apex (X,Y,Z)	0.000010, 0.000040, 0.000009	0.000023, 0.000041, 0.000056
Second Molar		
Crown (X,Y,Z)	0.000006, 0.000120, 0.000009	-0.000004, 0.000100, 0.000056
Apex (X,Y,Z)	0.000002, 0.000015, 0.000000	0.000023, 0.000020, 0.000055

In contrast, the Canine species exhibits changes largely along the Z-axis, where the apex without the canine orbital socket (COS) displays a negative displacement, as opposed to a positive displacement observed with the presence of the COS. The existence of a curve significantly affects the displacement of the apex, particularly in the Z direction, for the Second Premolar. However, the influence on the crown's displacements is minimal. The First Molar displays variations across all axes, but the shift from negative to positive values in the X direction for the crown with the COS stands out. Lastly, the Second Molar showcases the COS's impact, especially in the Z direction at the apex.

Table 2 provides insights into the displacement in the X, Y, and Z directions at an implant height of 13 mm with a force of 150 gm. For the Central Incisor, the crown displacement in the Y direction without the COS was 0.000110, which was reduced to 0.000093 with the COS. The Z-directional displacement at the apex showed a slight increase with the COS. In the case of the Lateral Incisor, the crown displacement in the X direction showed a negligible negative change with the COS, while the Z-directional displacement at the apex increased in the presence of the COS. 

**Table 2:** Displacement in X, Y, and Z direction at 13 mm implant height and 150 gm force

Type of teeth	Without Curve of Spee	With Curve of Spee
Central Incisor		
Crown (X,Y,Z)	0.000000, 0.000110, 0.000039	0.000000, 0.000093, 0.000100
Apex (X,Y,Z)	0.000000, 0.000043, 0.000076	0.000001, 0.000067, 0.000110
Lateral Incisor		
Crown (X,Y,Z)	0.000000, 0.000110, 0.000033	-0.000002, 0.000091, 0.000097
Apex (X,Y,Z)	0.000015, 0.000032, 0.000069	0.000022, 0.000055, 0.000110
Canine		
Crown (X,Y,Z)	0.000002, 0.000120, 0.000025	-0.000003, 0.000096, 0.000009
Apex (X,Y,Z)	0.000004, 0.000022, 0.000058	0.000013, 0.000048, 0.000099
Second Premolar		
Crown (X,Y,Z)	0.000005, 0.000100, 0.000007	-0.000001, 0.000090, 0.000076
Apex (X,Y,Z)	-0.000002,-0.000001, 0.000024	0.000011, 0.000016, 0.000083
FirstMolar		
Crown (X,Y,Z)	0.000010, 0.0000110, 0.000001	-0.000004, 0.000088, 0.000058
Apex (X,Y,Z)	0.000010, 0.000037, 0.000010	0.000023, 0.000039, 0.000059
Second Molar		
Crown (X,Y,Z)	0.000007, 0.000100, 0.000016	-0.000003, 0.000086, 0.000049
Apex (X,Y,Z)	-0.000001, 0.000014, 0.000007	0.000020, 0.000019, 0.000048

The Canine and Second Premolar displayed more pronounced changes in the X-direction, particularly at the crown. Generally, the introduction of the COS either maintained or slightly increased the Z-directional displacement across different teeth, especially at their apices. For the First Molar, crown displacement in the Y direction reduced with the COS. The Second Molar showed negative displacements in the X and Z directions without the COS but became less negative with its presence.

Table 3 presents the displacement data in the X, Y, and Z directions at an implant height of 16 mm when subjected to a 150-gm force. For the Central Incisor, introducing the COS resulted in a reduced Y-directional displacement at the crown while amplifying the Z-directional displacement. Interestingly, the apex showed a surge in Y-directional displacement in the presence of the COS, while the Z-displacement reduced slightly. The Lateral Incisor, when compared to its counterpart without the COS, exhibited reductions in Y-displacement for both crown and apex in the presence of the COS. Z-directional displacement, however, increased significantly in both sections of the tooth. The Canine's Z-directional displacement at the crown and apex showed similar amplification with the COS, while the Y-direction displacement generally decreased. In the Second Premolar and First Molar, the introduction of the COS resulted in lesser Y-directional displacements. Specifically, for the First Molar, the Y-displacement at the crown displayed a notable decrease. The Second Molar apex showed a considerable shift from a negative to a positive X-displacement with the COS.

**Table 3:** Displacement in X,Y,Z direction at 16 mm implant height and 150 gm force

Type of teeth	Without Curve of Spee	With Curve of Spee
Central Incisor		
Crown (X,Y,Z)	0.000000, 0.000087, 0.000100	0.000000, 0.000067, 0.000160
Apex (X,Y,Z)	0.000001, 0.000070, 0.000110	0.000001, 0.000094, 0.000140
Lateral Incisor		
Crown (X,Y,Z)	-0.000001, 0.000085, 0.000091	-0.000004, 0.000066, 0.000160
Apex (X,Y,Z)	0.000021, 0.000053, 0.000099	0.000028, 0.000076, 0.000140
Canine		
Crown (X,Y,Z)	-0.000001, 0.000093, 0.000074	-0.000005, 0.000073, 0.000140
Apex (X,Y,Z)	0.000011, 0.000041, 0.000085	0.000021, 0.000068, 0.000130
Second Premolar		
Crown (X,Y,Z)	0.000004, 0.000085, 0.000027	-0.000003, 0.000070, 0.000096
Apex (X,Y,Z)	0.000003, 0.000005, 0.00039	0.000016, 0.000022, 0.000098
First Molar		
Crown (X,Y,Z)	0.000009, 0.0000088, 0.000006	-0.000006, 0.000069, 0.000062
Apex (X,Y,Z)	0.000010, 0.000034, 0.000012	0.000023, 0.000035, 0.000061
Second Molar		
Crown (X,Y,Z)	0.000007, 0.000083, 0.000023	-0.000002, 0.000069, 0.000042
Apex (X,Y,Z)	-0.000004, 0.000013, 0.000014	0.000017, 0.000018, 0.000040

## Discussion

Anchorage is vital to the process of mass contraction, with significant emphasis placed on Newton's third law, also known as 'Newton's third law of motion'. This law recognizes that any action individuals perform will result in a response of equal magnitude but in the opposite direction [9]. It is common knowledge that retracting four incisors after canine retraction is a technique for reducing the forward advancement of the posterior tooth segment. In contrast, retracting all six anterior teeth at once may present difficulties in maintaining appropriate anchorage. A simultaneous retraction of the six anterior teeth, as opposed to a sequential retraction of the canine and four incisors, has the potential to shorten the duration of treatment and provide an earlier facial profile modification [10,11]. This occurrence increases patient adherence to treatment protocols. The ability of this method to precisely relocate molars and achieve the desired treatment outcome is a crucial component. The degree of maxillary retraction or dental displacement can be controlled by maintaining or discontinuing the retracting force [12]. En masse retraction is the recommended method; however, conventional mechanics are not used for en masse retraction to preserve anchoring. In this study, finite element models of the maxilla were generated, and orthodontic loading for en-masse retraction was simulated. Displacement patterns on the crown and root apex of teeth were evaluated by varying implant height (10 mm, 13 mm, and 16 mm) using 150 gms of force and (0.019x0.025" ss) wire with and without COS, utilizing 3D FEM.

FEA is a computational numerical technique utilized to solve intricate problems by breaking down complex systems into several interrelated elementary elements. The described approach is an approximation technique used for modeling deformations and 3D displacement distribution within bodies undergoing displacement. The initial procedure involves partitioning the intricate geometry into a group of smaller parts with limited dimensions. These elements collectively constitute the mesh model representing the structures under investigation. Each constituent can assume a distinct geometric configuration (e.g., triangle, square, tetrahedron, etc.) accompanied by a unique internal strain function. The equilibrium equation between the external forces exerted upon the element and the displacements seen at its nodes can be ascertained by employing these functions and considering the element's inherent geometry [10].

The reliability of analytical outcomes obtained from finite element models is greatly influenced by their construction, which requires their equivalence to actual objects in several respects. A representation with a higher number of nodes is needed to accurately represent reality. The process of generating increasingly advanced models demands significant effort and dedication. Nevertheless, the research of De Tolla et al. and Geng et al. indicates that finite element modeling provides a relatively accurate approach for displacement analysis despite these limitations and assumptions [13,14].

The micro-implants utilized in this investigation were of the self-drilling variety, measuring 1.6 millimeters in diameter and 8 millimeters in length and angulating at 90 degrees. Drill-free fasteners have greater bone contact than their pre-drilled counterparts. However, pre-drilling is recommended for the insertion of screws into thick, dense cortical bone [14]. According to Chen et al., self-drilling micro-implants can be employed on the maxilla, including the thin cortical bone portions from the jaw [15]. Drill-free implants displayed decreased mobility and increased histo-morphometric bone-metal contact. Drill-free implantation might produce less temperature change and bone debris. The impact of diameter on success rates was observed by Miyawaki et al., and Lim et al., but Park et al. found it to have no effect [16,17]. According to Lim et al., the mini screw's stability depends more on its diameter than its length [18]. Kyung et al. and Ishii et al. proposed the implementation of 8-mm long micro-implants for the maxilla, specifically placed between the patient's second maxillary premolar and their first permanent molar [19,20].

According to research by M. Jasmine and colleagues, increasing the insertion angles from 30 to 45 degrees, 60 degrees, and 90 degrees reduced micro-implant and cortical bone displacements [21]. Studies have also shown that for improved stability and effectiveness when placing a micro-implant within the posterior buccal area of the maxilla and the mandible, mini-screws are advised to be inserted 70 to 80 degrees into the long longitudinal axis of the teeth [22,23]. The greatest retention was achieved when temporary anchoring devices were positioned 90 degrees from the synthetic cortical plate. Therefore, a 90-degree angulation was employed in the current investigation to represent the most suitable clinical scenario [24]. A force of 150 grams was utilized in this study. The point of force application varied by changing the implant height (10 mm, 13 mm, and 16 mm) between the second premolar and the first molar to the attachment on the wire (0.019 × 0.025" SS), with and without the COS.

Displacement along the Y-axis (anteroposterior)

All teeth, including those with the COS, display distal tilting of the crown and apex. With increasing implant height, crown tipping decreases while apical tipping increases. Because force is transferred near the center of resistance, more bodily movement is observed at implant height. The angle developed among the force direction for the microscrew alongside the horizontal section is described as θ; modifying the θ could alter the biomechanical paradigm [25]. Increasing the implant's height would indeed increase the θ angle while decreasing the horizontal force. The horizontal force is equal to the force times cos θ. In our study, the maximal Y displacement is 10 mm, decreasing with height to a minimum of 16 mm at the same force level. These results are comparable to those of Hedayati and Shomali [25]. Changing the θ angle with ARH height may be inconvenient for the patient and clinically impossible, so it is preferable to change the implant height to control the force vector for displacement. Since implants are positioned apically to the crown at the proximal root surface, which can generate intrusive force vectors, a COS is incorporated into the wire to compensate for the intrusive component of force. In a previous FEA study, Sung et al. (2004) analyzed comparable outcomes with a curved arch wire [10,11].

Displacement in vertical (Z) direction(intrusion/extrusion)

The findings of the study revealed a vertical extrusion of 10 mm observed in all teeth at an implant height of 10 mm in the absence of Counter Ortho Stop (COS). The observed result can be attributed to the exertion of force at a location situated within the threshold of resistance. These findings are consistent with previous research conducted by Chetan S et al. [26] and Bohara et al. [27]. Previous studies conducted by Sung et al., Zhang et al., and Park et al. have provided evidence of central incisor intrusion. The variation in vertical displacement can be ascribed to the magnitude of the retraction force [10, 28, 29]. The estimated location of the central point of resistance for the six front teeth is equidistant between the resistance centers of the four incisors and the canine. More precisely, it is situated approximately 13.5 mm apically and 14 mm posterior to the incisal boundary of the upper central incisor [30-32]. When employing a Crown with Spee curve (CC), it was observed that each tooth had a degree of incursion at this particular height. The aforementioned result aligns with the conclusions drawn from the investigations carried out by Sung et al. in 2003 and 2010. In order to mitigate the constraints associated with the Anterior Repositioning Hook (ARH) CC, one potential approach may involve the implantation of an incisor wire into the maxillary arch [19,28].

However, the bracket slot and archwire will be redesigned to reduce the adhesion formed by the bracket slot and curved archwire, which can impede movement in the posterior slot. Consequently, we formulated a minimally compensating and clinically applicable curve. Each tooth's apex exhibited intrusion with and without COS. At implant heights of 13 mm and 16 mm, the crowns and apices of all teeth exhibited intrusion. Increasing the height of the implant increased the quantity of intrusion as the line of action moved closer to the center of resistance. As indicated by the force vector and the COS, the synergistic effect is greater with COS. The quantity of apical intrusion is greater anterior to the extraction space and nearly equal posterior to the implant site. This study was largely consistent with the findings of Kee-Joon et al. and Sung et al.. In higher implant height, the occlusal plane is rotated counterclockwise to align with the maxillary incisors and first molars. Sung and colleagues observed an intrusion [28,33].

Displacement in transverse (X) direction (medial/lateral)

In the transverse direction, all teeth exhibited medial/palatal/lingual movement, which is greater at the apex than at the crown, and the movement volume increases as implant height increases. As the archwire constrains the crown, more movement is observed in the apex than in the crown. Lateral incisor displacement was determined to be greater than central incisor displacement due to the deformation of the archwire at the site of placement of the retraction hook, which was distal to the lateral and distal to the canine. The identical pattern was noticed and reported by Sung et al., who used magnified FEM images of the region to illustrate the deformation [28].

On comparing displacement in all directions at various implant heights and the same force level, displacement varies with implant height. At a low implant height (10 mm), displacement is greatest along the sagittal direction, whereas at a high implant height (16 mm), displacement is greatest in the vertical direction. The sagittal and vertical dimensions are nearly identical on a medium (13 mm) implant. In the transverse direction, displacement is minimal regardless of implant height. Comparing implant heights of the same and various heights, the magnitude of displacement increases, which is accentuated with COS in the vertical direction attributable to the intrusive force component. Sung et al., Chetan et al., Upadhayay et al., Hedyati, and Shomali have investigated the effects of elevated placement implants. In addition to discussing en masse retraction, they alter the height of the anterior hook to modify the reaction force [25,26,28].

This study had some limitations in that the success of FEM analysis relies heavily on the accuracy of the models employed for the analysis; therefore, models must be built to be as close as possible to the real objects they represent. Biological variability is a consideration because these findings originate from a simulated model. The calculated values should be used merely as a guide to help inform medical judgment. However, the spatial and structural interactions between the various dentofacial components might vary from person to person. Recognizing that these variables may contribute to diverse responses of the dentofacial components to loading is essential.

## Conclusions

The outcomes derived from FEM underscore the sensitivity of results to model fidelity. While originating from simulated models, the study's findings stand as valuable references for clinical judgment. Recognizing individual variations in structural and spatial relationships among dentofacial components is crucial, given their contribution to diverse responses under loading. The significant impact of the COS on tooth displacements, notably affecting the Y and Z directions in different teeth, highlights the complexity of biomechanics. Ultimately, these insights enrich our understanding and guide informed clinical decision-making, emphasizing the multifaceted nature of the orthodontic treatment.
